# C-Terminal Modification Contributes the Antibacterial Activity of a Cecropin-like Region of Heteroscorpine-1 from Scorpion Venom

**DOI:** 10.3390/biology14081044

**Published:** 2025-08-13

**Authors:** Yutthakan Saengkun, Anuwatchakij Klamrak, Piyapon Janpan, Shaikh Shahinur Rahman, Rima Erviana, Nawan Puangmalai, Nisachon Jangpromma, Jureerut Daduang, Sakda Daduang, Jringjai Areemit

**Affiliations:** 1 Division of Pharmacognosy and Toxicology, Faculty of Pharmaceutical Sciences, Khon Kaen University, Khon Kaen 40002, Thailand; yutthakan_s@kkumail.com (Y.S.); anuwat_kla@yahoo.com (A.K.); j.piyapon@kkumail.com (P.J.); shahinanft@gmail.com (S.S.R.); sakdad@kku.ac.th (S.D.); 2 Protein and Proteomics Research Center for Commercial and Industrial Purposes (ProCCI), Khon Kaen University, Khon Kaen 40000, Thailand; nisaja@kku.ac.th (N.J.); jurpoo@kku.ac.th (J.D.); 3 Department of Applied Nutrition and Food Technology, Faculty of Biological Sciences, Islamic University, Kushtia 7000, Bangladesh; 4 School of Pharmacy, Universitas Muhammadiyah Yogyakarta, Jl. Brawijaya, Tamantirto, Bantul, Yogyakarta 55183, Indonesia; rima@umy.ac.id; 5 Innovative Pharma Herbs Co., Ltd., 67/3 M.5, Tha Raeng, Ban Laem 76110, Thailand; innovativeherb.nawan@gmail.com; 6 Department of Biochemistry, Faculty of Science, Khon Kaen University, Khon Kaen 40000, Thailand; 7 Centre for Research and Development of Medical Diagnostic Laboratories, Faculty of Associated Medical Sciences, Khon Kaen University, Khon Kaen 40002, Thailand; 8 Department of Pharmaceutical Technology, Faculty of Pharmaceutical Sciences, Khon Kaen University, Khon Kaen 40002, Thailand

**Keywords:** antimicrobial peptides, scorpion, heteroscorpine-1, end-tagging

## Abstract

The rise of multidrug-resistant pathogens has become a serious health concern, creating an urgent need for novel therapeutic approaches. Among the compounds explored, AMPs have emerged as promising candidates due to their broad-spectrum activity and low propensity for resistance development. However, their clinical implementation is limited by improper size, in vivo instability, and toxicity. Here, we designed short analogs of CeHS-1 via (1) truncation of intact CeHS-1, (2) amino acid substitution, (3) end-tagging, and (4) C-terminal amidation. The results showed that short analogs fused with an RWW stretch exhibited stronger antibacterial activity than the parent analogs, without inducing hemolysis in human red blood cells. Among the tested AMPs, mechanistic studies revealed membrane-disruptive activity of certain peptides against *Staphylococcus aureus*. In silico analyses also suggested that the analogs bind DNA by aligning parallel to its grooves, where the RWW stretch is believed to contribute to interactions between arginine and tryptophan residues and nitrogenous bases through electrostatic, hydrogen bonding, and hydrophobic interactions. The short CeHS-1 analogs established here may serve as potential alternative antimicrobial agents, which should be tested in clinical trials in the future.

## Simple Summary

Antimicrobial peptides (AMPs) are promising candidates for combating multidrug-resistant bacteria; however, the production of large peptides is often expensive. In this study, the cecropin-like region of heteroscorpine-1 (CeHS-1) was modified using truncation, amino acid substitution, end-tagging, and amidation strategies. The results demonstrated that end-tagging with an arginine–tryptophan–tryptophan (RWW) stretch enhanced the antibacterial activity and DNA-binding properties of short CeHS-1 analogs without causing damage to human red blood cells. Newly designed CeHS-1 analogs strongly interacted with the grooves of DNA, as shown by molecular docking and in vitro assays. Our study clearly shows that the RWW end-tag plays a crucial role in determining the antibacterial activity of short AMPs, as confirmed by the wet lab results.

## 1. Introduction

The global rise of multidrug-resistant (MDR) pathogens represents a critical public health emergency, with projections suggesting that antimicrobial resistance (AMR) could result in up to 10 million deaths annually by 2050 if left unmitigated [1,2,3]. This crisis is further exacerbated by a sharp decline in the development of new antibiotics [4]. As a result, alternative antimicrobial strategies are urgently needed. Antimicrobial peptides (AMPs), a group of naturally occurring defense molecules, have gained attention as promising candidates owing to their broad-spectrum efficacy, low cytotoxicity, and reduced risk of resistance development [5,6].

AMPs are typically composed of 10–50 amino acid residues and are produced by a wide array of organisms, including mammals, insects, amphibians, and plants [5,7,8,9]. These peptides play an integral role in innate immunity and display activity against bacteria, fungi, viruses, and protozoa [10,11]. Their antimicrobial function is largely attributed to their amphipathic structure and net positive charge ranging from +2 to +9, which enable selective interaction with negatively charged microbial membranes [12,13,14]. Hydrophobic domains promote membrane insertion and disruption through mechanisms such as pore formation and bilayer destabilization [15,16]. Moreover, AMPs may translocate across membranes and target intracellular components such as nucleic acids, further enhancing their bactericidal effect [5,17]. In addition to antimicrobial properties, certain AMPs have demonstrated anticancer potential, expanding their therapeutic applications [18].

Despite their therapeutic promise, AMPs face clinical translation challenges including proteolytic degradation, cytotoxicity, limited bioavailability, and high manufacturing costs [1,19]. To circumvent these limitations, rational design strategies such as sequence truncation, amino acid substitution, hydrophobic end-tagging, and C-terminal amidation have been employed to enhance peptide stability, potency, and specificity [20,21].

Short AMPs are particularly advantageous due to their ease of synthesis, structural simplicity, and favorable pharmacokinetics [22,23,24]. Notably, several truncated analogs of LL-37, such as P60.4Ac and SAAP-148, exhibit enhanced antibacterial activity and reduced toxicity, including efficacy against resistant pathogens [25,26]. Similarly, CeHS-1, derived from heteroscorpine-1 (HS-1) found in the venom of *Heterometrus laoticus*, has demonstrated potent antimicrobial activity, particularly against methicillin-resistant *Staphylococcus aureus* (MRSA) and multidrug-resistant *Klebsiella pneumoniae* [27].

Amino acid substitution is widely used to optimize AMP properties by modifying net charge, hydrophobicity, and amphipathicity [21,28,29]. Enhanced variants such as MEP-3 and MEP-4 outperform their parental peptide, melectin, in combating diverse bacterial strains [30]. Another optimization method, end-tagging with hydrophobic residues—especially stretches of tryptophan (Trp, W)—has been shown to improve peptide stability and antimicrobial potency [31,32,33]. Similarly, C-terminal amidation, a natural post-translational modification found in several animal-derived AMPs, neutralizes negative charges and boosts peptide–membrane interactions while protecting against carboxypeptidase-mediated degradation [34,35,36,37,38,39,40].

Venomous organisms such as scorpions represent a rich source of bioactive peptides, including AMPs with unique structural motifs and potent antimicrobial activity [41,42,43] CeHS-1, a 36-residue cecropin-like peptide located at the N-terminus of HS-1, possesses a typical cecropin structure composed of an amphipathic α-helical N-terminal region and a hydrophobic C-terminal segment. This peptide is active against both Gram-positive and Gram-negative bacteria, including resistant strains, while maintaining minimal hemolytic and cytotoxic activity [27]. However, large peptide size and associated production costs limit its broader application [44,45].

To address these challenges, the present study focused on the rational design of short CeHS-1 analogs by applying a systematic approach involving sequence truncation, targeted amino acid substitutions, end-tagging with an RWW tripeptide, and C-terminal amidation. The newly designed analogs were evaluated for their antibacterial efficacy, hemolytic activity, membrane disruption potential, and DNA-binding properties. The findings offer insight into the structure–activity relationships of short AMPs and highlight the therapeutic potential of cost-effective synthetic analogs derived from scorpion venom peptides.

## 2. Materials and Methods

### 2.1. Peptide Design

CeHS-1 was split at the hinge region (shown in Figure S1) into two fragments, F1-CeHS-1 and F2-CeHS-1. These fragments were then analyzed using the Antimicrobial Peptide Calculator and Predictor (available online at https://aps.unmc.edu/prediction) (accessed on 5 February 2023). The program revealed sequence homology between F1-CeHS-1 and F2-CeHS-1 and known potent AMPs. Each fragment was further divided at the midpoint, resulting in four shorter fragments: F1.1-CeHS-1, F1.2-CeHS-1, F2.1-CeHS-1, and F2.2-CeHS-1. These were subsequently compared to other short AMPs. Sequence alignment indicated a high degree of similarity between these smaller fragments and efficient short AMPs. Based on these results, the small fragments were used as templates for designing new CeHS-1 analogs.

The small fragments of CeHS-1 were analyzed for helical wheel projections and physicochemical properties using the HeliQuest service (http://heliquest.ipmc.cnrs.fr/cgi-bin/ComputParams.py) (accessed on 10 February 2023) to guide amino acid substitutions. Negatively charged residues (Asp and Glu) and some polar uncharged amino acids (Asn and Gln) were replaced with positively charged residues (Lys) and hydrophobic amino acids (Ile) to improve overall physicochemical properties and enhance amphipathic distribution in the helical wheel diagrams. Additionally, the hydrophobic and hydrophilic regions of the analogs were expanded by incorporating a tripeptide RWW stretch at the C-terminus. Furthermore, C-terminal amidation was performed to increase peptide stability against proteolytic degradation and to enhance antibacterial activity.

### 2.2. Peptide Synthesis

The modified CeHS-1 analogs were synthesized using Fmoc solid-phase peptide synthesis (SPPS) by GL Biochem (Shanghai) Ltd. (Shanghai, China) with a purity of ≥95%. The purity was further confirmed by reverse-phase high-performance liquid chromatography, and the molecular weight of the peptides was analyzed using mass spectrometry.

### 2.3. Antimicrobial Activity Assay

The following microorganisms were used to determine the minimum inhibitory concentration (MIC) and minimum bactericidal concentration (MBC) of the peptides: Gram-positive *Staphylococcus aureus* ATCC 25923, Gram-negative *Escherichia coli* ATCC 25922, *Klebsiella pneumoniae* ATCC 27736, and *Pseudomonas aeruginosa* ATCC 27853. The assays were conducted according to the protocol described by Erviana et al., with some modifications [27].

The antibacterial activity of the modified CeHS-1 analogs was evaluated using MIC and MBC assays. The MIC (minimum inhibitory concentration) is defined as the lowest concentration of antimicrobial peptides (AMPs) or other agents that inhibits visible bacterial growth. In contrast, the MBC (minimum bactericidal concentration) is defined as the lowest concentration of AMPs that kills 99.9% of the bacterial inoculum under the specified conditions [46]. The determination of MIC values was performed using a 96-well plate assay. Initially, peptides were serially diluted in the wells. Then, 10 µL of bacterial inoculum, previously adjusted to a cell density of 2.2 × 10^6^ CFU/mL, was added to each well. The plates were incubated at 37 °C for 16–18 h. The MIC was determined by measuring the optical density at 600 nm (OD_600_), with the lowest concentration of AMPs that inhibited visible bacterial growth defined as the MIC. For MBC determination, 25 µL of culture from wells showing no visible growth (i.e., those at and above the MIC) was spread onto nutrient agar (NA) plates and incubated at 37 °C for 16–18 h. The lowest concentration of AMPs at which no bacterial colonies were observed was defined as the MBC [47].

### 2.4. Hemolytic Activity Assay

The hemolytic activity of the peptides was assessed using human red blood cells (hRBCs), following the method described by Erviana et al., with some modifications [27]. Briefly, a 4% hRBC suspension was prepared in 1X phosphate-buffered saline (PBS, pH 7.4). Subsequently, 100 µL of the hRBC suspension was mixed with 100 µL of peptide samples at various concentrations (500, 200, 100, 50, and 25 µg/mL) in microcentrifuge tubes. The mixture was incubated at 37 °C for 1 h, followed by centrifugation at 100× *g* for 5 min. The supernatant was collected and transferred to a 96-well plate for absorbance measurement at 415 nm. The hemolytic activity of each peptide was subsequently calculated using the following equation:

$$Hemolysis\;(\%)=\frac{S}{P}\times 100$$

In the above equation, *S* represents the absorbance at 415 nm of the sample or negative control, while *P* represents the absorbance at 415 nm of the positive control. The peptide solvent was used as the negative control, whereas 0.1% Triton X-100 served as the positive control.

### 2.5. In Silico Prediction of Hemolytic Activity

The HAPPENN tool webserver (https://research.timmons.eu/happenn) (accessed on 10 April 2024) was used to predict the likelihood of AMP-induced hemolysis. The amino acid sequences of the effective peptides, including those with C-terminal amidation, were submitted to classify them as either hemolytic or non-hemolytic.

### 2.6. Membrane Disruption Property Assay

A bacterial suspension of *S. aureus* was cultured in LB medium to the exponential phase (OD_600_ = 0.5). The cell suspension was then diluted to 10^6^ CFU/mL in PBS and incubated with AMPs at varying concentrations (0.25, 0.5, and 1 MIC) for 1 h at 37 °C with continuous shaking at 180 rpm. Following incubation, 42 µL of SYTOX™ Green Ready Flow™ Reagent (Invitrogen, Thermo Fisher Scientific, Carlsbad, CA, USA) was added to the peptide-treated cells and incubated for an additional 10 min at 25 °C. Fluorescence emission was measured using flow cytometry. The cell population was gated based on forward scatter and side scatter parameters. Cells with compromised membranes were identified by their fluorescence (excitation at 488 nm and detection at 530 nm with a 30 nm bandpass filter). The mean fluorescence intensity and the percentage of fluorescent cells were determined by analyzing 100,000 cells per sample. Each experiment was performed in at least triplicate. Flow cytometry measurements were conducted using a BD FACSCanto II system (BD Biosciences, Wokingham, UK).

### 2.7. DNA-Binding Assay

Gel retardation assays were employed to examine the binding of CeHS-1 and its analogs to DNA, based on the method described by Anunthawan et al., with minor modifications. Briefly, plasmid DNA (pET32a vector) was mixed with 10× binding buffer (100 mM Tris–HCl, pH 8.0; 200 mM KCl; 10 mM EDTA; 10 mM DTT; and 50% (*v/v*) glycerol) and peptides at increasing peptide-to-DNA weight ratios of 0:1, 0.25:1, 0.5:1, 1:1, and 2:1. The mixtures were incubated at 25 °C for 1 h, after which 6× DNA loading dye was added. Samples were then loaded onto a 0.75% agarose/0.5× Tris-acetate-EDTA (TAE) gel and electrophoresed at 75 V for 1 h.

### 2.8. Molecular Docking

Docking analysis was performed using the HADDOCK 2.4 webserver (https://rascar.science.uu.nl/haddock2.4/) (accessed on 20 November 2024) to explore the binding interactions between AMPs and a DNA fragment. The HADDOCK analysis generated binding scores for the peptide–DNA complexes, calculated as a weighted sum of various energy terms, including van der Waals energy, electrostatic energy, desolvation energy, and restraints violation energy. The 3D structures of all peptides were generated using AlphaFold2 (https://colab.research.google.com/github/sokrypton/ColabFold/blob/main/AlphaFold2.ipynb) (accessed on 14 August 2024). The DNA fragment (a 20-base-pair sequence) was prepared from the 3D structure of the phage 434 Cro/OR1 complex (PDB: 3CRO) [48], with the phage 434 Cro protein removed using PyMOL. All amino acid residues and nucleotides were defined as active residues, indicating direct involvement in the interaction. Docking parameters were set to their default values. PyMOL and Discovery Studio were utilized to visualize and illustrate the interactions between the antimicrobial peptides (AMPs) and the DNA fragment.

## 3. Results

### 3.1. Peptides Design and Physicochemical Properties

F1-CeHS-1 and F2-CeHS-1, the fragments separated at the flexible hinge region of CeHS-1, shared high similarities with several small peptides classified as alpha-helix AMPs, as shown in Table 1. The comparison revealed similarities in the positioning of certain hydrophobic, positively charged, and other residues, which are highlighted in yellow, blue, and grey, respectively.

In addition, the shorter analogs of F1-CeHS-1 (F1.1-CeHS-1, F1.2-CeHS-1) and F2-CeHS-1 (F2.1-CeHS-1 and F2.2-CeHS-1) also exhibited strong similarities to several short AMPs, as detailed in Table 2 and Table 3. The amino acid alignment revealed resemblances in the positioning of specific hydrophobic and positively charged residues. This suggests that these peptide analogs have the potential to function as AMPs.

**Figure 1:**
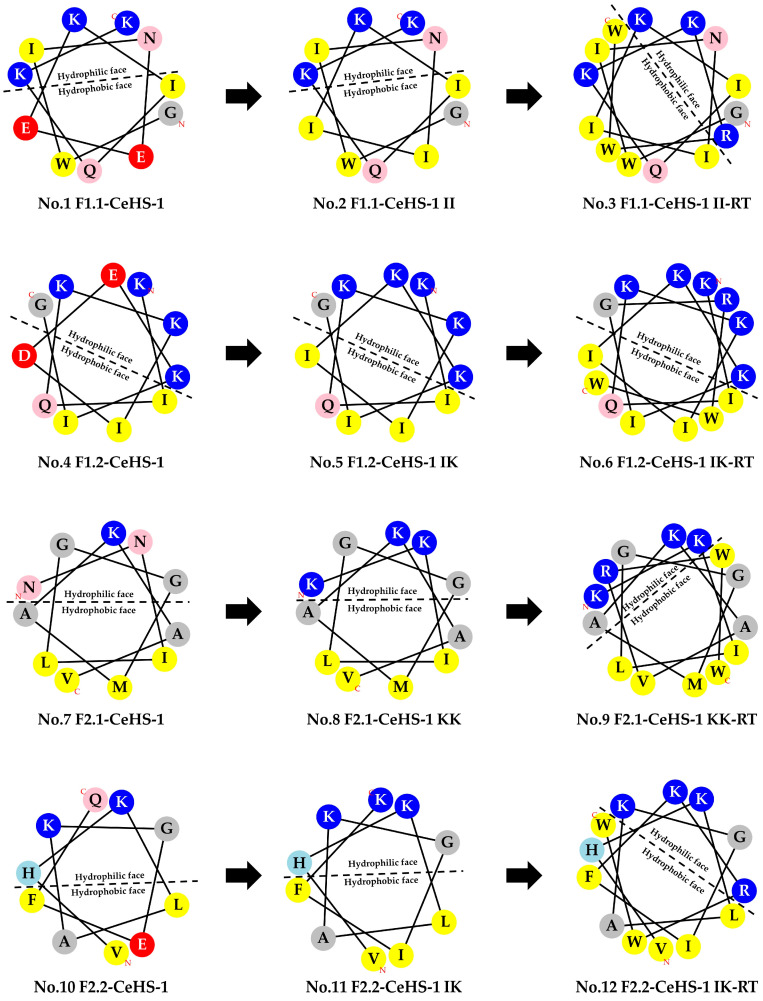
Helical wheel projections of CeHS-1 analogs.

To improve the short CeHS-1 analogs, the original peptides (F1.1-CeHS-1, F1.2-CeHS-1, F2.1-CeHS-1, and F2.2-CeHS-1) were modified through amino acid substitutions and C-terminal end-tagging with a RWW stretch. As shown in Table 4, these modifications enhanced the physicochemical properties of the peptides. Furthermore, addition of the RWW stretch at the C-terminus increased both the positive charge and hydrophobicity of the peptides. However, for peptide No. 3, this modification disrupted the amphipathic division, as illustrated in Figure 1.

### 3.2. Antimicrobial Activity

The modified CeHS-1 analogs (peptides No. 2, No. 3, No. 5, No. 6, No. 8, No. 9, No. 11, and No. 12) were evaluated for their antibacterial activities by determining the minimum inhibitory concentrations (MIC) and minimum bactericidal concentrations (MBC) against four bacterial strains. The MIC and MBC values in Table 5 reveal that the modified analogs with the added RWW stretch—specifically peptides No. 6, No. 9, and No. 12—exhibited inhibitory effects against all tested bacterial strains. In contrast, peptide No. 3 showed activity only against *E. coli*. The results also indicated that *E. coli* was most sensitive to peptide No. 12, while *K. pneumoniae* and *P. aeruginosa* showed the highest sensitivity to peptides No. 9 and No. 6, respectively. The parent peptides (peptide No. 2, No. 5, No. 8, and No. 11) showed no antibacterial activity against any of the tested strains.

### 3.3. Hemolytic Activity

**Figure 2:**
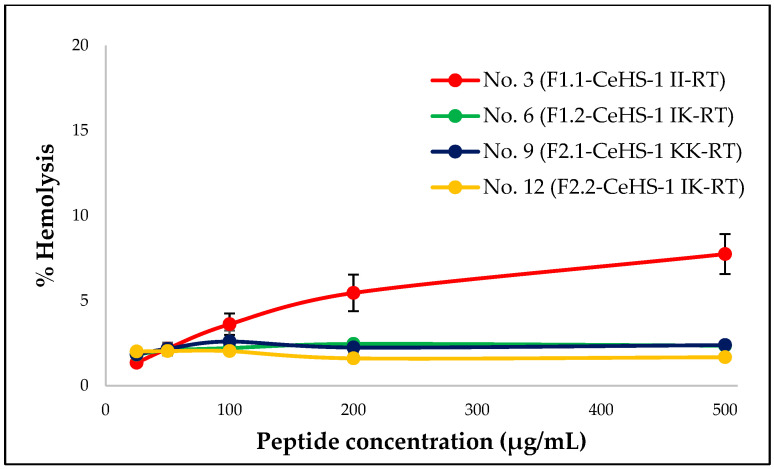
Hemolytic activity of CeHS-1 analogs against human red blood cells after 1 h exposure.

Toxicity is one of the key factors limiting the application of AMPs. To assess the safety of these peptides, the hemolytic activity of modified CeHS-1 analogs with potent antimicrobial properties was evaluated using human red blood cells (hRBCs). As shown in Figure 2, peptide No. 3 exhibited dose-dependent hemolytic activity, whereas the other peptides displayed consistent hemolytic properties across the tested concentration range. At the highest concentration tested (500 µg/mL), peptide No. 3 showed the greatest hemolytic activity, with a value of 7.74 ± 1.18%. In contrast, peptides No. 6, No. 9, and No. 12 exhibited lower hemolytic activities, with values of 2.35 ± 0.20%, 2.39 ± 0.22%, and 1.67 ± 0.17%, respectively. These results are almost consistent with the hemolytic predictions from HAPPENN, as shown in Table 6, where peptide No. 3 had the highest PROB score (0.556), indicating a greater likelihood of causing hemolysis. Although, peptides No. 6 and No. 12 were predicted to have low PROB scores, they still exhibited similar average hemolytic activity to peptide No. 9, which had a PROB score of 0.262 based on the results, a score over 20 times greater than those of peptides No. 6 and No.12. This indicates that care must be taken when interpreting results. The computational analysis is useful for initial screening, while should be complemented by experimental validation to ensuring the accuracy of the findings.

### 3.4. Membrane Disruption Property

Many AMPs kill bacteria by inducing membrane disruption and causing leakage of cellular contents [69,70]. Therefore, we evaluated the ability of CeHS-1 analogs (No. 6, No. 9, and No. 12) to disrupt the cell membrane integrity of *S. aureus*—a well-known pathogen associated with a range of skin infections, including atopic dermatitis, impetigo, and wound infections [33]—using a fluorescence dye-based assay with the DNA-binding probe SYTOX™ Green. SYTOX™ Green is a high-affinity nucleic acid stain that becomes fluorescent only upon binding to nucleic acids and cannot penetrate intact cell membranes. This property enables the distinction between non-permeabilized and permeabilized cells by detecting increased fluorescence when the dye binds to DNA [70,71,72]. Ampicillin and kanamycin were used as negative control compounds, as these antimicrobial agents exert their effects without disrupting the bacterial cell membrane [73,74].

**Figure 3:**
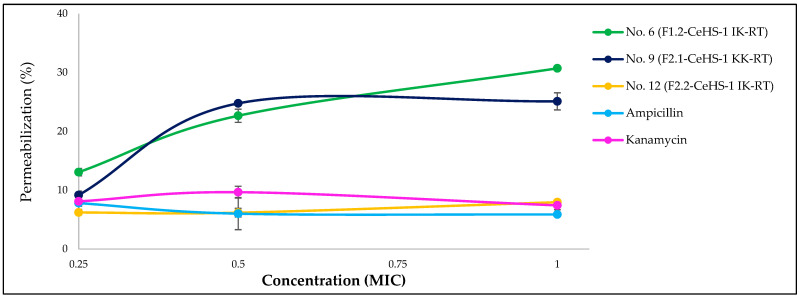
Cytoplasmic membrane permeabilization of *S. aureus* evaluated from SYTOX™ Green fluorescence emission intensity.

The results presented in Figure 3 reveal that peptides No. 6 and No. 9 disrupt the cytoplasmic membrane of *S. aureus* in a dose-dependent manner, as indicated by increased fluorescence intensity. In contrast, peptide No. 12 exhibited consistently low fluorescence intensity, similar to that observed with the negative control compounds, ampicillin and kanamycin.

### 3.5. DNA-Binding Property 

**Figure 4:**
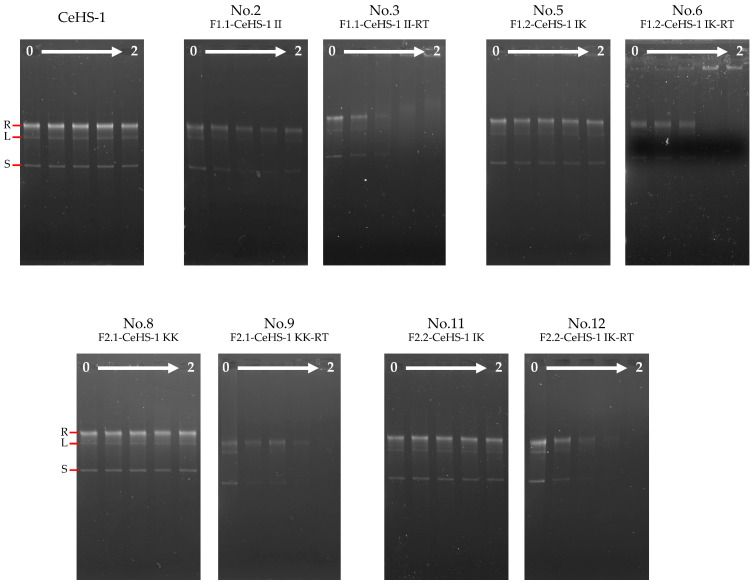
Peptide binding to plasmid DNA was assessed using a 0.75% agarose/TAE gel, with varying peptide-to-plasmid weight ratios. The ratios tested were 0:1, 0.25:1, 0.5:1, 1:1, and 2:1, displayed from left to right. The first lane, labeled ‘0’, represents the negative control with no peptide added. R represents the position of the relaxed circular plasmid, L denotes the linear plasmid, and S indicates the supercoiled plasmid.

As previously mentioned, AMPs can penetrate bacterial cell membranes and bind to intracellular targets, particularly DNA, thereby disrupting DNA replication and RNA synthesis, which ultimately leads to cell death [5,17]. Thereby DNA could be another target of inhibition. To prove this hypothesis, the DNA-binding abilities of CeHS-1 and its analogs were evaluated using plasmid DNA (pET32a vector) as the target. The results presented in Figure 4 indicate that peptides incorporating the RWW stretch, specifically peptides No. 3, No. 6, No. 9, and No. 12, effectively inhibited DNA migration at a 1:1 plasmid DNA-to-peptide weight ratio. In contrast, CeHS-1 and the other peptides did not interfere with DNA migration, even at the highest weight ratio tested. These findings highlight that incorporating the RWW stretch significantly enhances the DNA-binding properties of the peptides. Furthermore, the results demonstrate that these peptides can bind to multiple forms of plasmid DNA.

### 3.6. Molecular Docking for DNA-Binding Property

Molecular docking studies were performed using the HADDOCK 2.4 webserver to elucidate the interactions between DNA and the CeHS-1 analogs. As shown in Table 7, CeHS-1 analogs lacking the RWW stretch (No. 2, No. 5, No. 8, and No. 11) exhibited binding scores ranging from −37.6 to −54.4 kcal/mol, which are higher than those of Magainin 2, a peptide known for its weak DNA-binding properties. In contrast, CeHS-1 analogs containing the RWW stretch (No. 3, No. 6, No. 9, and No. 12) demonstrated lower binding scores, ranging from −69.3 to −80.0 kcal/mol. These values are lower than those of Magainin 2 and are comparable to RT2 and KT2, antimicrobial peptides known to bind bacterial DNA to exert their effects. The results suggest that incorporating the RWW stretch at the C-terminus significantly enhances the DNA-binding ability of the peptides, while analogs lacking this modification exhibit weaker binding. This finding is consistent with the gel retardation assay results.

**Figure 5:**
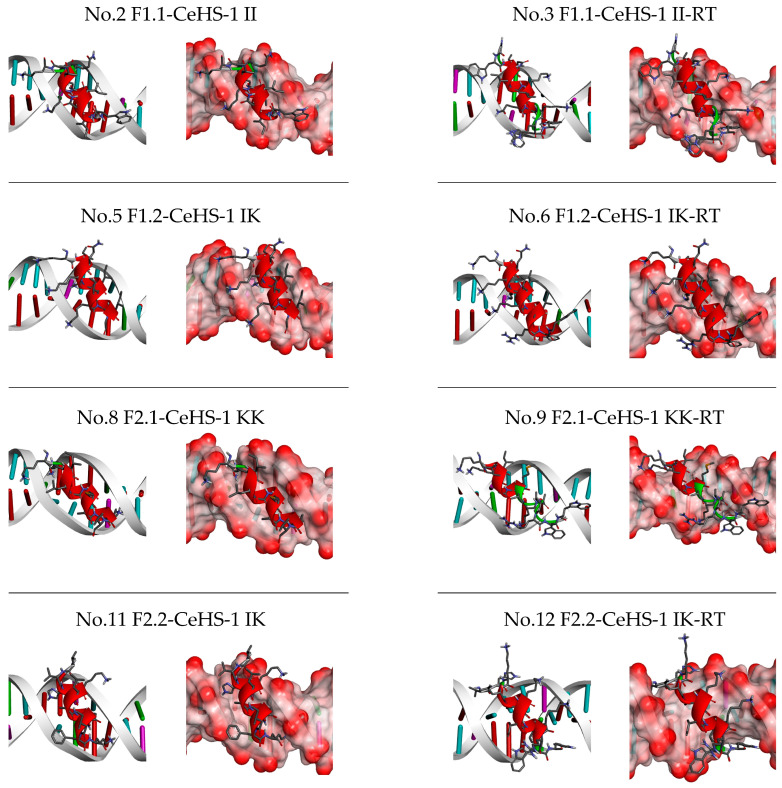
Illustrates a hypothetical interaction between CeHS-1 analogs and DNA fragment.

In addition, molecular docking analysis (Figure 5) revealed binding interactions between the peptide and DNA, with the peptide aligning parallel to the grooves of the DNA structure. Further interaction analysis (Table 7) showed that positively charged amino acids (Lys, Arg, and His) mainly interacted with the phosphate groups through hydrogen bonding and electrostatic interactions. Conversely, hydrophobic aromatic amino acids such as Trp and Phe primarily interacted with the nitrogenous bases via hydrogen bonding and hydrophobic interactions. These findings suggest that adding the RWW stretch at the C-terminus enhances the DNA-binding ability of CeHS-1 by increasing interactions with both the nitrogenous bases and the phosphate backbone of the DNA structure.

## 4. Discussion

Although CeHS-1 demonstrates potent antimicrobial properties, its full-length structure poses challenges in pharmaceutical development due to high production costs associated with synthetic manufacturing. While recombinant expression in *Escherichia coli* has been explored as an alternative production method, this strategy has yielded limited success due to reduced antimicrobial activity and low peptide yield [75]. In response to these challenges, this study employed rational peptide engineering to design shorter analogs of CeHS-1 that retain or exceed the activity of the parent peptide while minimizing cytotoxic effects and reducing synthesis costs.

Generally, the amino acid sequences of AMPs can be divided into two regions—hydrophobic and hydrophilic—that are typically of similar size. Many studies have attempted to improve the antibacterial activity of peptides by tagging them with hydrophobic oligopeptides, such as Trp and phenylalanine (Phe, F) [32,33]. Building on this strategy, Anunthawan et al. developed a systematic approach to enhance antibacterial activity of Luecrocin I isolated from the white blood cells of *Crocodylus siamensis* by simultaneously introducing both hydrophobic and hydrophilic regions through the incorporation of specific amino acid stretches and found exceptional enhanced antimicrobial activities of the modified peptide. In their design (ABBABBAABB/B), “A” denotes positively charged amino acids (e.g., Lys), while “B” denotes hydrophobic amino acids (e.g., Typ). However, both CRT2 and CRT3 demonstrated approximately threefold-higher hemolytic toxicity compared with the original Luecrocin I, likely due to the increased hydrophobicity resulting from the incorporation of these long stretches [31]. This bring us to use the RWW stretch—the shortest repeating amino acid motif—as it is more suitable for our objective of developing a systematic approach to designing short peptides and minimizing the risk of hemolytic activity associated with high hydrophobicity.

The systematic approach employed—comprising sequence truncation, amino acid substitution, C-terminal RWW end-tagging, and amidation—resulted in several analogs with enhanced antibacterial activity and physicochemical properties. The addition of the RWW tripeptide stretch significantly improved both the net positive charge and hydrophobicity of the analogs (as shown in Table 4), thereby promoting bacterial membrane interaction and disruption. These findings align with previous research demonstrating that the spatial arrangement of hydrophobic and cationic residues is critical for AMP efficacy [31,32]. However, the structural integrity and amphipathic division of the peptide remain essential, as evidenced by peptide No. 3, which exhibited lower antimicrobial activity despite RWW end-tagging—likely due to an unfavorable amphipathic profile.

Among the analogs, peptides No. 6, No. 9, and No. 12 emerged as the most promising candidates, demonstrating broad-spectrum antimicrobial activity at low MIC values (4–32 µg/mL) without inducing hemolysis in human red blood cells. These analogs thus satisfy key pharmacological criteria, including selectivity toward microbial membranes and minimal toxicity to host cells. Computational predictions using the HAPPENN tool further validated their low hemolytic potential, offering a cost- and time-efficient strategy for future AMP screening.

Mechanistic assays provided insight into the dual mode of action exhibited by these analogs. Peptides No. 6 and No. 9 disrupted bacterial membranes in a dose-dependent manner, as evidenced by increased SYTOX Green fluorescence, indicative of cytoplasmic membrane permeabilization. Interestingly, peptide No. 12 did not exhibit significant membrane-disruptive effects but instead displayed strong DNA-binding activity, suggesting an intracellular target-based mechanism. These findings are consistent with previous studies indicating that AMPs can act via both membrane-lytic and intracellular modes, including interference with DNA replication and transcription [5,17,76].

Gel retardation and molecular docking analyses confirmed the DNA-binding capabilities of the RWW-tagged peptides. Docking scores for peptides No. 6, No. 9, and No. 12 were comparable to known DNA-targeting AMPs such as RT2 and KT2. The RWW motif appeared to facilitate multiple electrostatic and hydrophobic interactions with phosphate backbones and nitrogenous bases, thus stabilizing peptide–DNA binding. These interactions highlight the structural and functional advantages of RWW incorporation, providing a framework for future AMP design.

Despite these promising results, large-scale production remains a hurdle. While chemical synthesis is feasible for research and clinical testing, it is not cost-effective for mass production. As such, developing a microbial expression system for the production of these short CeHS-1 analogs is a logical next step. This approach could offer advantages in scalability, speed, and cost, especially if combined with downstream purification strategies optimized for short, cationic peptides [77,78].

In summary, this study underscores the potential of rationally designed short AMPs as viable alternatives to traditional antibiotics, particularly in the context of rising drug resistance. By combining structural bioinformatics, in vitro assays, and molecular docking, this study presents a robust model for peptide optimization that can be extended to other AMP scaffolds.

## 5. Conclusions

In this study, we successfully designed small CeHS-1 analogs through a combination of sequence shortening, amino acid substitution, end-tagging with an appended RWW stretch, and C-terminal amidation. As demonstrated, the short CeHS-1 analogs containing the RWW stretch (peptides No. 3, No. 6, No. 9, and No. 12) exhibited promising antibacterial activity across various pathogenic bacteria while inducing no hemolysis in hRBCs. These analogs demonstrated DNA-binding ability, as suggested by molecular docking, and possibly displayed membrane-disruptive activity against *S. aureus*. Furthermore, our study suggests that the addition of the RWW stretch significantly enhances the antibacterial activity and DNA-binding capacity of CeHS-1 analogs by improving their physicochemical properties via enhanced positive charge and hydrophobicity.

## Figures and Tables

**Table 1:** Sequence alignment of CeHS-1 and its analogs with AMPs.

Peptide	Origin	Sequence																																																						AA	Identity (%)	APD ID	Ref.
CeHS-1		G	W	I	N	E	E	K	-	I	Q	K	K	I	D	E	K	-	I	G	N	N	I	L	G	-	-	-	G	-	-	-	M	A	-	K	-	A	V	-	V	H	K	-	L	A	K	G	-	-	E	-	F	-	Q	37			
F1-CeHS-1	This work	G	W	I	N	E	E	K	-	I	Q	K	K	I	D	E	K	-	I	G																																							
Aurein 1.1	*Litoria aurea*	G	-	-	-	L	F	D	-	I	I	K	K	I	A	E	S	-	I	-																																				13	41.18	AP00012	[49]
P11-5	*Synthetic peptide*	G	-	-	-	-	-	K	-	L	F	K	K	I	L	-	K	-	I	L																																				11	41.18	AP03683	[50]
Mastoparan-AF	*Anterhynchium flavomarginatum micado*	-	-	I	N	L	L	K	-	I	A	K	G	I	I	-	K	-	S	L																																				14	41.18	AP01517	[51]
P11-6	*Synthetic peptide*	-	-	-	-	-	K	K	L	I	-	K	K	I	L	-	K	-	I	L																																				11	38.89	AP03684	[50]
Hylaseptin P1	*Hyla punctata*	G	-	I	-	L	D	A	-	I	A	-	K	I	A	-	K	A	A	G																																				14	38.89	AP01249	[52]
Spiniferin	*Heterometrus spinifer*	-	-	I	L	G	E	-	-	I	W	K	G	I	-	-	K	D	I	L																																				13	38.89	AP02551	[53]
F2-CeHS-1	This work																				N	N	I	L	G	-	-	-	G	-	-	-	M	A	-	K	-	A	V	-	V	H	K	-	L	A	K	G	-	-	E	-	F	-	Q	21			
Maximin 31	*Bombina maxima*																				-	G	I	-	G	-	-	-	G	A	L	L	S	A	G	K	S	A	-	-	-	L	K	G	L	A	K	G	L	A	E	H	F	-	-	25	43.33	AP01732	[54]
Bombinin	*Bombina variegata L*																				-	G	I	-	-	-	-	-	G	A	L	-	S	A	-	K	G	A	-	-	-	L	K	G	L	A	K	G	L	A	E	H	F	A	N	24	42.86	AP00049	[55]
Nigrocin-1-OW3	*Odorrana wuchuanensis*																				-	G	I	L	G	N	I	V	G	-	-	-	M	-	G	K	K	-	V	-	V	C	G	-	L	-	S	G	L	C						21	41.67	AP01883	[56]
Ocellatin-P1	*Leptodactylus pentadactylus*																				-	G	L	L	D	T	L	K	G	-	-	-	A	A	-	K	N	-	V	-	V	-	G	S	L	A	S	K	V	M	E	-	-	L	-	25	40.00	AP00540	[57]
Phylloseptin-H11	*Phyllomedusa hypochondrialis*																				F	L	S	L	-	-	I	P	-	-	-	-	H	A	I	-	N	A	V	G	V	H	-	-	-	A	K	-	-	-	-	H	F	-	-	19	39.13	AP00972	[58]
Uperin 3.6	*Uperoleia inundata*																												G	V	I	-	A	A	-	K	K	-	V	-	V	N	V	-	L	-	K	N	L	-	-	-	F			17	38.10	AP00325	[59]

The yellow, blue, and grey backgrounds indicate the conserved hydrophobic, positively charged, and other amino acid residues, respectively.

**Table 2:** Sequence alignment of F1-CeHS-1 and its analogs with AMPs.

Peptide	Origin	Sequence																							AA	Identity (%)	APD ID	Ref.
F1-CeHS-1		G	-	W	-	I	N	E	E	K	-	I	Q	K	K	I	D	E	-	-	K	-	I	G	17			
F1.1-CeHS-1	This work	G	-	W	-	I	N	E	E	K	-	I	Q	K	K										11			
IK2	Synthetic peptide	G	-	-	-	I	-	I	K	K	-	I	I	K	K	I									10	50.00	AP04801	[60]
B6	Synthetic peptide	G	I	W	S	D	L	A	E	-	-	I	I	K	K	F									13	42.86	AP03851	[61]
Halictine 2	*Halictus sexcinctus*	G	K	W	-	M	S	L	L	K	-	I	L	K											12	38.46	AP01923	[62]
F1.2-CeHS-1	This work								-	K	-	I	Q	K	K	I	D	E	-	-	K	-	I	G	11			
IK2	Synthetic peptide								-	G	-	I	I	K	K	I	I	K	-	-	K	-	I	-	10	54.55	AP04801	[60]
P11-6	Synthetic peptide								K	K	L	I	-	K	K	I	-	L	-	-	K	-	I	L	11	53.85	AP03684	[50]
Hp1036	*Heterometrus petersii*								-	-	-	I	L	G	K	I	W	E	G	I	K	S	I	F	13	42.86	AP02334	[63]

The yellow, blue, and grey backgrounds indicate the conserved hydrophobic, positively charged, and other amino acid residues, respectively.

**Table 3:** Sequence alignment of F2-CeHS-1 and its analogs with AMPs.

Peptide	Origin	Sequence																																		AA	Identity (%)	APD ID	Ref.
F2-CeHS-1		N	N	I	-	-	L	-	G	-	-	G	-	M	A	-	-	K	A	V	V	H	K	-	L	-	-	A	K	-	G	E	F	Q	-	21			
F2.1-CeHS-1	This work	N	N	I	-	-	L		G	-	-	G	-	M	A	-	-	K	A	V																11			
Temporin-SN2	*Hylarana spinulosa*	-	F	I	T	G	L	I	G	-	-	G	L	M	-	-	-	K	A	L																13	46.67	AP02273	[64]
Temporin-SN3	*Hylarana spinulosa*	-	F	I	S	G	L	I	G	-	-	G	L	M	-	-	-	K	A	L																13	46.67	AP02274	[64]
OdMa4	*Odorrana margaretae*	-	G	I	-	-	L	S	G	L	L	G	-	-	A	G	K	K	I	V	C															15	41.18	AP03539	[65]
F2.2-CeHS-1	This work																				V	H	K	-	L	-	-	A	K	-	G	E	F	Q	-	10			
PN5	*Pinus densiflora*																				-	F	K	F	L	-	-	A	R	T	G	K	F	L		11	41.67	AP03449	[66]
Gramicidin S	*Bacillus brevis*																				V	-	K	-	L	F	P	V	K	-	-	L	F	Q	-	10	41.69	AP02243	[67]
MD4K	*Myxine glutinosa* L.																			G	I	H	K	I	L	-	-	-	K	Y	G	K	P	S	-	12	38.46	AP04009	[68]

The yellow, blue, and grey backgrounds indicate the conserved hydrophobic, positively charged, and other amino acid residues, respectively.

**Table 4:** Amino acid sequences and physicochemical properties of amidated CeHS-1 analogs.

No.	Peptide Name	Amino Acid Sequence	AA	Net Charge *	%H	H	µH
1	F1.1-CeHS-1	GWINEEKIQKK-NH_2_	11	+2	22.27	0.071	0.215
2	F1.1-CeHS-1 II	GWINIIKIQKK-NH_2_	11	+4	45.45	0.515	0.497
3	F1.1-CeHS-1 II-RT	GWINIIKIQKKRWW-NH_2_	14	+5	50.00	0.654	0.461
4	F1.2-CeHS-1	KIQKKIDEKIG-NH_2_	11	+3	22.27	−0.017	0.609
5	F1.2-CeHS-1 IK	KIQKKIIKKIG-NH_2_	11	+6	36.36	0.185	0.691
6	F1.2-CeHS-1 IK-RT	KIQKKIIKKIGRWW-NH_2_	14	+7	42.86	0.394	0.814
7	F2.1-CeHS-1	NNILGGMAKAV-NH_2_	11	+2	54.55	0.398	0.575
8	F2.1-CeHS-1 KK	KKILGGMAKAV-NH_2_	11	+4	55.55	0.327	0.618
9	F2.1-CeHS-1 KK-RT	KKILGGMAKAVRWW-NH_2_	14	+5	57.14	0.506	0.622
10	F2.2-CeHS-1	VHKLAKGEFQ-NH_2_	10	+2	40.00	0.231	0.394
11	F2.2-CeHS-1 IK	VHKLAKGIFK-NH_2_	10	+4	50.00	0.398	0.697
12	F2.2-CeHS-1 IK-RT	VHKLAKGIFKRWW-NH_2_	13	+5	53.85	0.575	0.654

Green, red, and blue represent the original amino acids, substituted amino acids, and tripeptide stretches, respectively. %H: hydrophobic amino acid content, H: hydrophobicity, µH: hydrophobic moment. * Amidation at the C-terminus increases the net charge of a peptide by one positive unit (+1).

**Table 5:** Determination of MIC of CeHS-1 analogs.

No.	Peptide Name	*S. aureus*		*E. coli*		*K. pneumoniae*		*P. aeruginosa*	
		MIC	MBC	MIC	MBC	MIC	MBC	MIC	MBC
2	F1.1-CeHS-1 II	ND	ND	ND	ND	ND	ND	ND	ND
3	F1.1-CeHS-1 II-RT	ND	ND	128	256	ND	ND	ND	ND
5	F1.2-CeHS-1 IK	ND	ND	ND	ND	ND	ND	ND	ND
6	F1.2-CeHS-1 IK-RT	32	64	8	8	256	256	16	32
8	F2.1-CeHS-1 KK	ND	ND	ND	ND	ND	ND	ND	ND
9	F2.1-CeHS-1 KK-RT	64	128	8	32	128	128	64	128
11	F2.2-CeHS-1 IK	ND	ND	ND	ND	ND	ND	ND	ND
12	F2.2-CeHS-1 IK-RT	32	32	4	16	256	256	32	64
	Positive control								
	Ampicillin	8	8	4	32	>256	>256	128	256

MIC: minimum inhibitory concentration. MBC: minimum bactericidal concentration. MIC and MBC values are determined in µg/Ml. ND indicates no detection at the highest tested concentration.

**Table 6:** Potential hemolytic activity of CeHS-1 analogs identified using HAPPENN tool.

No.	Peptide Name	PROB
3	F1.1-CeHS-1 II-RT	0.556
6	F1.2-CeHS-1 IK-RT	0.007
9	F2.1-CeHS-1 KK-RT	0.262
12	F2.2-CeHS-1 IK-RT	0.010

The PROB score is a normalized sigmoid score ranging from 0 to 1. A score of 0 is predicted to be most likely non-hemolytic, and a score of 1 is predicted to be most likely hemolytic.

**Table 7:** Binding scores (in kcal/mol) and molecular interaction of CeHS-1 analogs and DNA fragment.

No	Peptide Name	Binding Energy (Kcal/mol)	RMSD	Chemical Bond Interaction (Å)/Nucleotide Composition		
				H-Bonds ● Conventional H-Bond ● Carbon H-Bond ● Pi-Donor H-bond	Charge ● Salt Bridge; Charge-Charge ● Charge-Charge ● π-Cation ● π-Anion	Hydrophobic Interactions
						● Alkyl ● π-alkyl ●π-π shaped
2	F1.1 CeHS-1 II	−37.6 ± 4.5	−5.2 ± 1.0	Trp2●P/●P, Ile3●D, Lys7●P/●P, Lys10●G/●T/●T/●D, Lys11●P	Trp2●P, Lys7●P/●P, Lys11●P	N.D
3	F1.1 CeHS-1 II-RT	−71.8 ± 3.5	−9.0 ± 0.8	Lys7●P, Gln9●P, Lys10●D/●A/●C/●T	Trp2●●P, Lys7●P/●P, Lys11●P/●P,	Trp14●T
5	F1.2-CeHS-1 IK	−47.3 ± 10.9	−7.4 ± 0.6	Lys5●●T, Lys9●A/●C/●A/●D, Ile10●D	Lys1●P/●P, Lys4●P	N.D
6	F1.2-CeHS-1 IK-RT	−80.0 ± 6.3	−8.7 ± 0.1	Lys5●T/●D, Lys9●C/●T, Arg12●P, Trp13●P, Trp14●P	Lys1●●P, Lys4●P, Lys8●P/●P, Arg12●●●P/●●P,	N.D
8	F2.1-CeHS-1 KK	−41.7 ± 12.7	−2.3 ± 1.4	Lys1●P, Lys2●C/●P/●D, Gly6●D, Lys9●T/●C/●C/●●D	Lys1●●P/●P	N.D
9	F2.1-CeHS-1 KK-RT	−79.4 ± 7.3	−5.9 ± 0.3	Lys1●P, Lys2●T/●A, Lys9●A	Lys1●P/●●P, Lys2●C/●P, Lys9●P/●P, Arg12●P/●P, Trp14●P	Lys2●●A/●A, Lys9●A
11	F2.2-CeHS-1 IK	−42.8 ± 3.7	−5.0 ± 0.9	His2●T, Lys6●T/●G/●D, Lys10●T/●D/●P	Val1●P, Lys3●●P/●P, Phe9●P	N.D
12	F2.2-CeHS-1 IK-RT	−69.3 ± 2.9	−4.7 ± 0.9	His2●P, Lys6●P, Lys10●P, Trp13●A	Val1●P, His2●P, Lys6●P/●P, Lys10●P/●P, Trp12●●P	Val1●A, Ala5●●A, Phe9●A/●A, Trp13●●A/●A
Positive control						
	RT2	−81.7 ± 15.0	1.0 ± 0.9			
	KT2	−90.5 ± 14.6	2.1 ± 1.2			
Negative control						
	Magainin 2	−59.4 ± 9.9	10.6 ± 0.5			

N.D.: not detected, P: phosphate group, D: deoxyribose sugar, nitrogenous base; A: Adenine, G: Guanine, C: Cytosine, and T: Thymine.

## Data Availability

All data supporting the conclusions of this article are included in this article.

## Supplementary Materials

The following supporting information can be downloaded at https://www.mdpi.com/article/10.3390/biology14081044/s1. Figure S1: 3D structure of CeHS-1. Table S1: The amino acid sequences of the AMPs mentioned in the Introduction and Discussion sections [25,26,27,29,30].
