# Supplementary material for: C-Terminal Modification Contributes the Antibacterial Activity of a Cecropin-like Region of Heteroscorpine-1 from Scorpion Venom
Source: Biology (Basel). 2025 Aug 13;14(8):1044. doi: 10.3390/biology14081044 (PMC12383718; doi:10.3390/biology14081044)
Supplement: Supplementary file 1 [file biology-14-01044-s001.zip › biology-3744725-supplementary.pdf]

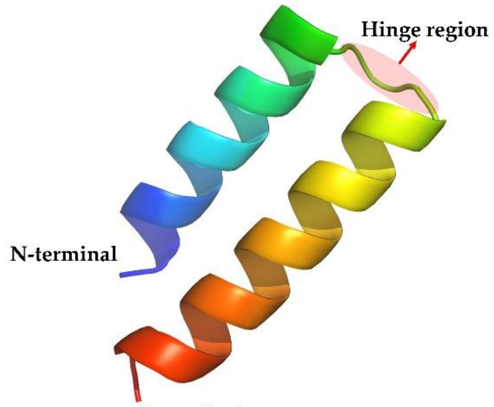

Figure S1. 3D structure of CeHS-1

Table S1. The amino acid sequences of the AMPs mentioned in the Introduction and Discussion sections

Peptide name	Sequence	Ref.
LL-37	LLGDFFRKSKEKIGKEFKRIVQRIKDFLRNLVPRTES	[25]
P60.4Ac	IGKEFKRIVERIKRFLRELVRPLR	
SAAP-148	LKRVWKRVFKLLKRYWRQLKKPVR	[26]
HS-1	GWINEEKIQKKIDEKIGNNILGGMAKAVVHKLAKGEFQCVANIDTMGNCETH CQKTSGEKGFCHGTKCKCGKPLSY	[27]
Melectin	GFLSILKKVLPKVMAHMK-NH_2	[29]
MEP-3	GFLSILKKVLAKVMAHMK-NH_2	
MEP-4	GFLSILKKVLGKVMAHMK-NH_2	
Leucrocin I	NGVQPKY	[30]
CRT2	NGVQPKYRWWRWWRRWW	
CRT3	NGVQPKYRWWRWWRRWWW	
